# Chloroform in Obstetrics

**Published:** 1860-09

**Authors:** Wm. Pettigreu

**Affiliations:** Western Med. and Surg. Society


### CHLOROFORM IN OBSTETRICS.

BY WM. PETTIGREU, WESTERN MED. AND SURG. SOCIETY.

As a general rule he deprecated its use in ordinary or natural labor, for the following reasons:

1. That the accoucher should attend solely and strictly to his own avocation, and that it therefore necessitates the presence of a second practitioner.

2. The folly of incurring any risk of asphyxia or death, although such cases in the lying-in room are rare with chloroform in comparison with those in which surgical operations are performed.

3. That under ordinary circumstances where matters are favorable and progress natural, it tends to depress the system leaving the entire expulsion of the fœtus to the efforts of the uterus, supplied, as it is, by organic nerves, while the muscles of animal life which so forcibly assist its action are almost paralysed.

4. By its administration there is danger both to the mother and child.

Cases illustrative of these objections were related; the exception to the general rule being, where the mother was of a delicate and nervous temperament, and where chloroform was administered in a very modified form, more to attract the attention of the patient from her fears than to lessen the natural throes of labor.

In protracted labor the author had experienced much benefit in its administration, and although the pains for the first ten minutes appeared arrested, they afterwards returned more strongly, with greater regularity, and under its use the rigidity relaxed, the mucous became more freely secreted, the countenance of the patient became less anxious, and the pulse quickened at first, became stronger, and the child was born in a very short time. Cases illustrative of these facts were then related.

The author, in his limited experience, bore out the observation of Dr. Simpson, that hemorrhage seldom or never occurred after the use of chloroform.—*Medical Times and Gazette.*

